# Identification of lung cancer with high sensitivity and specificity by blood testing

**DOI:** 10.1186/1465-9921-11-18

**Published:** 2010-02-10

**Authors:** Petra Leidinger, Andreas Keller, Sabrina Heisel, Nicole Ludwig, Stefanie Rheinheimer, Veronika Klein, Claudia Andres, Andrea Staratschek-Jox, Jürgen Wolf, Erich Stoelben, Bernhard Stephan, Ingo Stehle, Jürg Hamacher, Hanno Huwer, Hans-Peter Lenhof, Eckart Meese

**Affiliations:** 1 Department of Human Genetics, Medical School, Saarland University, Building 60, 66421 Homburg/Saar, Germany; 2 Center for Bioinformatics, Saarland University, Building E.1.1, 66041 Saarbruecken, Germany; 3 LIMES (Life and Medical Sciences Bonn), Genomics and Immunoregulation, University of Bonn, 53115 Bonn, Germany; 4 Department I of Internal Medicine, Center for Integrated Oncology, University Hospital Cologne, 50937 Cologne, Germany; 5 Lung clinic, Hospital of Cologne, 51109 Cologne, Germany; 6 Department of Clinical Haemostaseology and Transfusion Medicine, Medical School, Saarland University, Building 75, 66421 Homburg/Saar, Germany; 7 Department of Pneumology, Medical School, Saarland University, Building 91, 66421 Homburg/Saar, Germany; 8 Department of Pneumology, Inselspital, 3010 Bern, Switzerland; 9 Department of Cardiothoracic Surgery, Voelklingen Heart Center, 66333 Voelklingen/Saar, Germany

## Abstract

**Background:**

Lung cancer is a very frequent and lethal tumor with an identifiable risk population. Cytological analysis and chest X-ray failed to reduce mortality, and CT screenings are still controversially discussed. Recent studies provided first evidence for the potential usefulness of autoantigens as markers for lung cancer.

**Methods:**

We used extended panels of arrayed antigens and determined autoantibody signatures of sera from patients with different kinds of lung cancer, different common non-tumor lung pathologies, and controls without any lung disease by a newly developed computer aided image analysis procedure. The resulting signatures were classified using linear kernel Support Vector Machines and 10-fold cross-validation.

**Results:**

The novel approach allowed for discriminating lung cancer patients from controls without any lung disease with a specificity of 97.0%, a sensitivity of 97.9%, and an accuracy of 97.6%. The classification of stage IA/IB tumors and controls yielded a specificity of 97.6%, a sensitivity of 75.9%, and an accuracy of 92.9%. The discrimination of lung cancer patients from patients with non-tumor lung pathologies reached an accuracy of 88.5%.

**Conclusion:**

We were able to separate lung cancer patients from subjects without any lung disease with high accuracy. Furthermore, lung cancer patients could be seprated from patients with other non-tumor lung diseases. These results provide clear evidence that blood-based tests open new avenues for the early diagnosis of lung cancer.

## Background

Lung cancer is the leading cause of cancer death worldwide. In 2008, lung cancer is estimated to account for more than 200,000 new cancer cases and 160,000 cancer deaths in the United States [1]. Independent of the histological subtype, the five-year survival rate is among the lowest of all cancers.

Patients with stage IA and IB NSCLC (non small cell lung cancer) show 5-year survival rates between 60 to 80%, and patients with stage IIA and IIB NSCLC between 40 to 50% [2]. The 5-year relative survival rate varies markedly depending on the stage at time of diagnosis, from 49 to 16 to 2% for local, regional, and distant stage disease, respectively [3]. More than two thirds of lung cancer tumors are diagnosed at late stages when the survival rate is low [4].

As for other cancers, these data suggest that catching lung cancer while it is still small and locally defined significantly increases the chances of a cure [4]. Recent evidence shows that low dose spiral computed tomography (CT) detects lung cancer at smaller sizes and earlier than chest X-ray (CXR) that failed to identify 79% of lung cancers that were smaller than 2 cm [5]. A major drawback of low dose CT is the large number of false positive tests and the diagnosis of indolent tumors which in turn leads to an increased morbidity from unnecessary surgical treatment [6-8].

Molecular testing offers an opportunity for cancer detection before the occurrence of histopathological changes. The immune response to the tumor may recognize altered proteins of the tumor *in situ *and/or proteins that have been shed by the tumor and are circulating in the bloodstream. Recently, we identified a complex humoral immune response that could be used for the early diagnosis of brain cancer [9]. As of recently, however, only limited data were available on the identification and validation of serum markers for the diagnosis of lung cancer. Single serum markers showed only a low sensitivity and specificity for the identification of lung cancer patients [10-15]. Several studies with smaller panels encompassing few markers including known autoantigens, cancer associated proteins, and serum proteins like GAGE7, p53, HER2, CEA or alpha1-antitrypsin provided first evidence that simultaneous analysis of several antigens have a higher potential for separating patients with lung carcinoma from controls [16-19]. Using a panel of 82 phage peptide clones we were able to separate squamous cell lung carcinoma from control sera with an accuracy of 93%. Low-grade squamous cell lung carcinoma were distinguished from control sera with an accuracy of 92.9% [20]. Here, we extended our analysis by using an increased number of newly identified antigens to analyze not only squamous cell lung carcinoma but also other NSCLC as well as SCLC (small cell lung cancer). Furthermore, as a novelty we implemented and employed an image analysis method for evaluating the seroreactivity of the arrayed antigens.

Our study was to address the following questions: Can immunogenic antigens be used to differentiate lung cancer patients from controls with a very high sensitivity and specificity? Can stage I lung carcinomas be separated from controls without any lung disease with a comparable high sensitivity and specificity? Can lung cancer patients be separated from patients with other non-tumor lung pathologies (NTLP)? The affirmative answers to these questions will lay the basis for a prospective study aimed towards early identification of lung carcinomas.

## Methods

### Patients' sera

Blood samples of lung cancer patients were obtained from the Department of Pneumology of the Saarland University, from the Department of Cardiothoracic Surgery, SHG Clinic Voelklingen/Saar, and from the Cologne Smoking Study (CoSmoS), University Hospital of Cologne. The patient sera stem from 29 NSCLC patients, and from 18 SCLC patients. The mean age of NSCLC patients was 64.0 years (ranging from 45 to 83 years), and the mean age of SCLC patients was 60.4 years (ranging from 49 to 74 years). More detailed information of lung cancer patients is given in Table 1.

**Table 1:** Clinical parameters of the lung cancer patients

	NSCLC	SCLC
Total number, n (%)	29 (61.7)	18 (38.3)
- adenocarcinoma, n (%)	11 (37.9)	
- large cell carcinoma, (%)	9 (31.0)	
- squamous cell carcinoma, n (%)	9 (31.0)	
age mean, years (range)	64.00 (45-83)	60.44 (49-74)
male, n (%)	16 (55.2)	9 (50)
female, n (%)	13 (44.8)	9 (50)
		
**Clinical staging, n (%)**		
I	21 (72.4)	1 (5.6)
II	2 (6.9)	0 (0)
III	5 (17.2)	13 (72.2)
IV	0 (0)	2 (11.1)
unknown	1 (3.4)	2 (11.1)

*Abbreviations: *NSCLC = non-small cell lung cancer, SCLC = small cell lung cancer

As one control group we combined 80 sera from different volunteers without lung cancer or without other NTLPs (in the following designated as "controls" or "control sera"). Out of these 80 controls we obtained 60 sera from healthy blood donors from the Department of Clinical Haemostaseology and Transfusion Medicine (Saarland University, Homburg) and 20 sera from patients with diseases not affecting the lungs (e.g. slipped disc or myocardial infarction) from the Cologne Smoking Study (CoSmoS). As a second control group we used 26 sera of patients with common non-tumor lung pathologies (NTLP), i.e. 20 patients with COPD/emphysema, and 6 patients with pneumonia. The NTLP sera were obtained from the Department of Pneumology (Saarland University, Homburg), and from the Department of Cardiothoracic Surgery, SHG Clinic Voelklingen/Saar. The control sera as well as the NTLP sera were not matched for age and gender. The mean age of all control and NTLP patients was 46.5 years, ranging from 21 to 74 years. The smoking status of the blood donors was unknown. All blood samples were obtained with patients' informed consent. Serum was isolated from the blood samples and stored as aliquots at -70°C.

### Protein macroarray screening

High-density protein arrays consisting of 38,016 *E. coli *expressed proteins from the hex1 cDNA expression library [21] were screened with sera from patients with various human diseases including tumors and inflammatory diseases. In total, 1827 peptide clones that were reactive in this primary screening were combined on customized macroarrays and screened with 47 lung cancer sera, 26 NTLP sera, and 80 control sera. Screening was performed as described previously [22]. In brief, macroarrays were washed twice with TBSTT (TBS, 0.05% Tween 20, 0.5% Triton X-100) and 4 times with TBS. After incubation in blocking solution (3% nonfat dry milk powder in TBST (TBS, 0.05% Tween 20)), macroarrays were incubated over night with sera 1:1000 diluted in blocking solution. After the incubation, sera were stored for the second incubation round. Three washing steps with TBST were followed by incubation with stripping solution at 70°C. Macroarrays were washed twice with TBST and 4 times with TBS. Incubation with blocking solution was followed by the second round of serum incubation over night. Macroarrays were washed three times with TBST, and incubated with secondary antibody (rabbit anti-human IgG, IgA, IgM-Cy5 (H+L)) 1:1000 diluted in blocking solution. Macroarrays were washed four times with TBST, twice with TBS and scanned by the GE Healthcare Typhoon 9410 scanner, with 50 μm resolution, and 300 PMT.

### Image Analysis of protein macroarrays

We have developed an image analysis pipeline that ensures a standardized evaluation of the macroarrays. In a preprocessing step, the scanned images were adjusted, i.e., slight rotations of the scanned images were corrected and the edges of the image were virtually cut. Then, the array was segmented into regular subgrids that were in turn divided into the so-called spot (target) areas containing exactly one protein spot. By k-means clustering, the pixels belonging to the spot area of a protein were divided into foreground and background pixels. Optionally, the computed spot areas were adjusted, if the spot pixels exceeded the originally calculated target area. For the extraction of the dark protein spots a morphological operator from image processing, the so-called black top hat, utilizing a square structuring element has been applied to the image [23]. Finally, the intensity of each spot was calculated as the mean value of all pixels of the spot in the processed image. For each protein array, the automated analysis provided the intensities of all protein spots. Since each expressed protein was spotted in duplicates on the macroarray, the mean intensity of the two replicates was assigned to each protein. Thus, the image analysis of each macroarray resulted in an autoantibody profile consisting of 1827 integer intensity values ranging from 0 to 255, the standard range of values in a grey scale image.

### Statistical evaluation of autoantibody profiles

To minimize inter-array-effects, the measured autoantibody profiles that consist of the intensity values of the 1827 proteins were normalized using quantile normalization.

For the different classification tasks, we used standard linear kernel Support Vector Machines (SVMs) [24] with cost C of 1 that were evaluated by applying 10-fold cross validation. The classification procedure was repeated 10 times and the median of sensitivity, specificity, and accuracy was calculated. To test the model for overfitting, we further validated our prediction approach by performing so-called permutation tests, i.e., we randomly permuted class labels and performed classifications with the randomized data.

To directly access the "value" of an antigen with respect to its ability to separate two serum groups (1 and 2) from each other, we calculated the area under the Receiver Operator Characteristics curve (AUC) for each antigen *A *as follows: the normalized intensities of all analyzed sera were used as threshold values. For all thresholds *t*, we considered sera from group 1 with intensity value above *t *as true positives (TP), sera from group 1 with intensity value below *t *as false negatives (FN), sera from group 2 with intensity value below *t *as true negatives (TN), and sera from group 2 with intensity value above *t *as false positives (FP). Likewise for all thresholds, specificity (TN/(TN+FP)) and sensitivity (TP/(TP+FN)) were computed. Please note that in some cases the classification has to be inverted. In these cases, sera from group 1 with intensity value below *t *are considered as 'true positives' (TP). The Receiver Operator Characteristics (ROC) curve shows the sensitivity as function of one minus the specificity. AUC values can range from 0 to 1. An AUC of 0.5 for a spot means that the distribution of intensity values of sera from group 1 and sera from group 2 can not be distinguished. The more the AUC value of an antigen differs from 0.5, the better this antigen is suited to separate between the two serum groups. AUCs of 1 or 0 correspond to a perfect separation. Antigens with AUC values < 0.5 show higher intensity values in sera from group 1 than in sera from group 2. Antigens with AUC values > 0.5 show higher intensity values in sera from group 2 than in sera from group 1 [22].

## Results

To identify a larger number of proteins associated with an autoantibody response we screened high-density protein macroarrays generated from the fetal brain cDNA expression library hex1 encompassing more than 38,000 different peptide clones [21]. This primary screening included sera from patients with various human diseases including tumors and inflammatory diseases. In total, we identified 1827 peptide clones that reacted with serum autoantibodies. We arrayed these peptide clones and screened the resulting biomarker array with sera from 29 NSCLC patients, 18 SCLC patients, 26 patients with common NTLPs, and 80 controls without lung cancer or other lung pathologies. For each array, we calculated the normalized spot intensities and AUC values of all antigens as detailed in Material and Methods.

Based on these autoantibody profiles we studied ten different classification problems. Besides the separation of lung cancer versus controls, we built prediction models for low-grade lung tumors (stage I A/I B) versus controls, lung cancer stage II, III, IV versus controls, lung cancer stage IA/IB versus lung cancer stage II, III, IV, NSCLC versus controls, SCLC versus controls, NSCLC versus SCLC, lung cancer versus NTLP, lung cancer versus COPD/emphysema, and lung cancer versus non-cancer (80 controls and 26 NTLP). To this end, we applied linear kernel SVMs evaluated by 10 repetitions of 10-fold cross validation.

**Figure 1:**
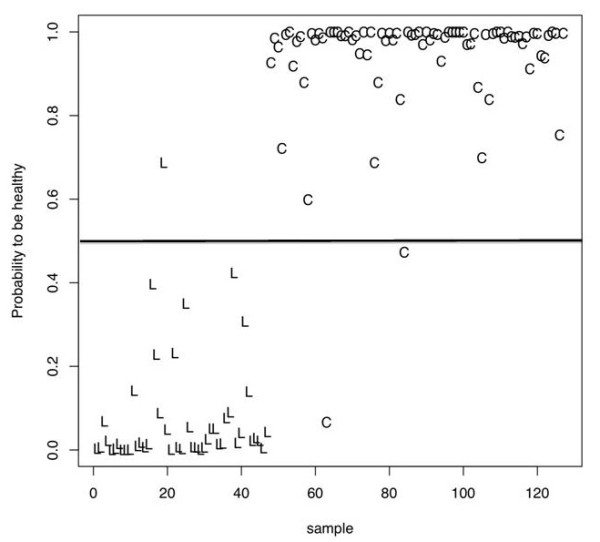
**Estimated probabilities for each serum to be a control sample**. Here, each 'C' corresponds to serum of patients without any lung disease and each 'L' to a lung cancer serum. The horizontal black line defines the classification threshold.

With our peptide clone set, we were able to differentiate lung cancer sera and control sera with an accuracy of 97.6%. In detail, we reached a sensitivity of 97.9% and a specificity of 97.0% (see Figure 1). Given the specificity of 97.0% and a total of 80 normal sera, we have a False Positive rate of 3.0%, i.e., only two of the 80 normal samples have been predicted to be lung cancer samples by cross-validation. The False Negative rate of 2.1% on the 47 lung cancer samples means that on average only one cancer sample has been predicted to be a normal sample. Taken together, three of all 127 samples have been wrongly classified while 124 samples have been correctly predicted.

The small standard deviation (0.007) of the classification accuracy demonstrates the stability of classification results. To test the model for overfitting, we carried out 100 permutation tests in a stratified manner that yielded a median accuracy of 54.3%. This can be explained by the differences in the class sizes. Permutation tests with even label distributions lead to an average accuracy of approximately 50%. Additionally, we carried out classification in stage I A/I B lung cancer sera and control sera. This comparison reached a sensitivity of 75.9%, a specificity of 97.6%, and an accuracy of 92.9%.

Next, we asked whether our peptide clone set also permits to discriminate between the two different histological lung cancer subgroups NSCLC and SCLC. While we were not able to distinguish between NSCLC and SCLC with very high accuracy (67.3%), we succeeded in separating NSCLC sera from control sera with a sensitivity of 97.8%, a specificity of 85.7%, and an accuracy of 94.6%. The classification of SCLC versus controls reached a sensitivity of 99.3%, a specificity of 79.4%, and an accuracy of 95.7%. As further controls, we used sera from patients with other non-tumor lung pathologies, e.g. COPD/emphysema and pneumonia. We were able to distinguish lung cancer patients from patients with NTLPs with an accuracy of 88.5%. We combined all non-cancer sera (80 sera of blood donors without any lung disease and the 26 sera of patients with NTLPs) and calculated the classification lung cancer versus non-cancer. Here, we reached an accuracy of 92.2%, a sensitivity of 96.7%, and a specificity of 88.2%. The classification lung cancer versus COPD/emphysema reached an accuracy of 78.8%, a sensitivity of 92.6%, and a specificity of 40.9%. For all ten classification tasks, permutation tests showed a significantly decreased performance (Wilcoxon Mann-Withney p-value < 10^-10^). All classification results and the 95% Confidence Intervals (CI) are summarized in Table 2. The AUC values for all ten classification tasks and each antigen are given in Additional file 1.

**Table 2:** Summary of the results of the ten different classification tasks.

Classification task	Accuracy %	Sensitivity %	Specificity %
lung cancer vs controls	97.6 (CI: 97.3-97.9)	97.9 (CI: 96.6-97.8)	97.0 (CI:97.6-98.3)
lung cancer stage IA/IB vs controls	92.9 (CI: 92.4-93.4)	75.9 (CI: 73.7-78.2)	97.6 (CI: 97.3-97.8)
lung cancer stage II, III, IV vs controls	97.6 (CI: 97.1-98.0)	99.9 (CI: 87.2-91.0)	89.1 (CI: 99.7-100.0)
lung cancer stage IA/IB vs lung cancer stage II, III, IV	66.5 (CI: 63.8-69.1)	65.7 CI: 62.2-69.2)	67.3 (CI: 64.6-70.0)
NSCLC vs controls	94.6 (CI: 93.9-95.2)	97.8 (CI: 97.5-98.2)	85.7 (CI: 83.8-87.5)
SCLC vs controls	95.7 (CI: 95.2-96.0)	99.3 (CI: 99.0-99.6)	79.4 (CI: 77.5-81.4)
NSCLC vs SCLC	67.3 (CI: 65.4-69.3)	76.7 (CI: 74.4-79.0)	52.2 (CI: 50.0-54.4)
lung cancer vs NTLP	88.5 (CI: 88.0-89.3)	99.8 (CI: 99.5-100.0)	42.4 (CI: 40.2-44.6)
lung cancer vs non-cancer	92.2 (CI: 91.6-92.9)	96.7 (CI: 95.5-97.9)	88.2 (CI: 87.5-88.8)
lung cancer vs COPD/emphysema	78.8 (CI: 78.0-79.7)	92.6 (CI: 91.6-93.5)	40.9 (CI: 38.8-43.0)

*Abbreviations: *NSCLC = non-small cell lung cancer, SCLC = small cell lung cancer, NTLP = non-tumor lung pathology, controls = patients without any lung disease, non-cancer = controls and NTLP, CI = 95% Confidence Intervals

## Discussion

There is widespread consensus that detection of cancer at an early stage can be live saving. Lung cancer screening however remains a controversial issue since CXR and sputum cytology screening showed no benefit towards reduced mortality [6,8]. Low dose CT can detect tumors that are smaller in size and are at an earlier stage in development. However, the high sensitivity of low dose CT screening is balanced by a rather high rate of false positive scans of up to 20% [25], which ultimately prove benign after unnecessary surgical interventions [26]. In summary, screening of persons asymptomatic for lung cancer is not recommended at present neither by CXR, sputum cytology nor by low dose CT [27].

Nevertheless, the identification of small lung cancers had lead to renewed interest in more specific markers that may contribute to an early identification of lung cancer. While the immune system is not efficient in destroying lung cancer, it recognizes cancer cells and can be utilized as diagnostic tool: Autoantibodies against cancer-associated antigens can be measured up to 5 years before symptomatic disease [16], have a long half-life [28], and can be detected at low costs. Single autoantigens that are immunogenic in lung cancer show a low sensitivity and specificity [10-15]. Combined measures of antibody reactivities to a panel of several arrayed proteins have been used to discriminate sera of lung cancer patients from control sera. These proteins include known tumor associated antigens like p53 and c-myc, known serum proteins like carcinoembryonic antigen and apha1-antitrypsin, and five predictive phage-expressed proteins recognized by serum antibodies [16-19]. There was no overlap between the protein sets used in these studies indicating a large number of immunogenic proteins that still await identification.

Lung carcinoma show complex cellular abnormalities including multiple genetic changes, like DNA sequence alterations, copy number changes, and aberrant methylation [29-32]. Extended antigen panels may most adequately analyze this cellular heterogeneity. The examination of a large antigen panel that reflects the complexity of immunogenic alterations in tumor cells likely contributes to a reliable classification with high accuracy, sensitivity, and specificity. The disadvantage of screening complex cDNA expression libraries with a large number of sera is, however, an inevitable degree of variability due to expression variability of bacterial *in vivo *expression systems. Nevertheless, in this study we were able to differentiate lung cancer patients from controls with high accuracy. Likewise, we were able to separate lung cancer patients from patients with non-tumor diseases of the lung with high accuracy.

Future studies have to elucidate whether the analysis of subgroups of lung cancer will be possible by smaller antigen panels. Smaller panels will facilitate to use full-length purified proteins for diagnostic purposes at affordable costs. Ideally, such antigen sets will even render the separation of histopathological subgroups like SCLC and NSCLC feasible.

The usefulness of complex antigen patterns for early lung cancer diagnosis awaits further confirmation by prospective studies. The reduction of the lung cancer associated mortality is an independent problem. There is an evident correlation between tumor size and survival time [4] for lung cancer. However, some lung cancers metastasize while the original tumor is still small. It is supposed that metastasis appears to primarily depend on the tumor genetics and the angiogenesis. In summary, our study shows that the immune system, even though not effective in destroying the tumor, may very well provide highly sensitive and specific markers for lung cancer in its early development.

## Conclusions

Autoantibodies are potentially well suited as cancer biomarkers, because only a minimal-invasive intervention is needed for their extraction, they can be easily measured, they are stable in blood, and they have a long half-life. The drawback of single autoantibodies is their low diagnostic sensitivity. Here, we show that the combination of an expanded number of autoantigens for the creation of complex autoantibody profiles allow for the differentiation of lung cancer patients from patients with other common non-tumor lung diseases or healthy blood donors with high accuracy.

## List of abbreviations used

NTLP: non-tumor lung pathology; NSCLC: non-small cell lung cancer; SCLC: small cell lung cancer; SVM: Support Vector Machine; AUC: area under the receiver operator characteristics curve; CI: Confidence Interval; TP: true positives; TN: true negatives; FP: false positives; FN: false negatives; CXR: chest X-ray; CT: computed tomography; COPD: chronic obstructive pulmonary disease.

## Competing interests

The authors declare that they have no competing interests.

## Authors' contributions

PL and EM conceived the study. PL, SH, NL, SR, and VK carried out the macroarray screening. AK, CA, and HPL were in charge of all computational and statistical analysis of the protein macroarrays. PL, AK, EM, and HPL wrote the manuscript. HH, JH, IS, BS, ASJ, JW, and ES supplied all tested sera.

## Supplementary Material

Additional file 1 **Summary of the AUC values for all ten classification tasks and each analyzed antigen.** Antigens considered as informative (AUC value < 0.3 or > 0.7) were written in red. Click here for file
